# The evaluation of tooth whitening from a perspective of artificial intelligence: a comparative analytical study

**DOI:** 10.3389/fdgth.2025.1710159

**Published:** 2025-11-24

**Authors:** Alaa Al-Haddad, Mikel Alrabadi, Othman Saadeh, George Alrabadi, Yazan Hassona

**Affiliations:** 1 School of Dentistry, The University of Jordan, Amman, Jordan; 2 School of Medicine, The University of Jordan, Amman, Jordan

**Keywords:** tooth whitening, dental bleaching, AI, large language models, patient education, cosmetic dentistry

## Abstract

**Background:**

Artificial intelligence (AI) chatbots are increasingly consulted for dental aesthetics information. This study evaluated the performance of multiple large language models (LLMs) in answering patient questions about tooth whitening.

**Methods:**

109 patient-derived questions, categorized into five clinical domains, were submitted to four LLMs: ChatGPT-4o, Google Gemini, DeepSeek R1, and DentalGPT. Two calibrated specialists evaluated responses for usefulness, quality (Global Quality Scale), reliability (CLEAR tool), and readability (Flesch-Kincaid Reading Ease, SMOG index).

**Results:**

The models generated consistently high-quality information. Most responses (68%) were “very useful” (mean score: 1.24 ± 0.3). Quality (mean GQS: 3.9 ± 2.0) and reliability (mean CLEAR: 22.5 ± 2.4) were high, with no significant differences between models or domains (*p* > 0.05). However, readability was a major limitation, with a mean FRE score of 36.3 (“difficult” level) and a SMOG index of 11.0, requiring a high school reading level.

**Conclusions:**

Contemporary LLMs provide useful and reliable information on tooth whitening but deliver it at a reading level incompatible with average patient health literacy. To be effective patient education adjuncts, future AI development must prioritize readability simplification alongside informational accuracy.

## Introduction

Tooth whitening has become an increasingly popular cosmetic procedure globally, influenced by social media and celebrity notions of the “Hollywood smile”. Recent data indicate a rising demand among patients for whiter teeth; for instance, over one million Americans undergo whitening treatments annually, generating approximately $600 million in dental revenue (1, 2). Market analyses estimate that the global teeth whitening industry was worth nearly USD 6.9 billion in 2021, with projections toward USD 10.6 billion by 2030 (3).

Consumers now have access to a variety of whitening methods, including in-office bleaching, over-the-counter kits, and home remedies, reflecting a global trend toward improved dental aesthetics. Simultaneously, patients frequently turn to the internet to seek health-related information. In the United States, 58.5% of adults reported using the internet to search for health or medical information within a recent year (4). Notably, Artificial Intelligence (AI)-driven chatbots (such as OpenAI's ChatGPT and Google's Gemini) provide patients with round-the-clock interactive guidance on medical topics via smartphones and web browsers (5–7). These advanced large language model (LLM) chatbots are trained on extensive text corpora, allowing them to generate coherent, human-like responses to user queries (8, 9). Consequently, AI chatbots have become a convenient source of dental information, effectively making critical oral health advice readily accessible to patients (7).

AI chatbots have experienced rapid development. OpenAI's ChatGPT (with GPT-3.5 introduced in 2022 and GPT-4 in 2023) and Google's Gemini (launched in 2023) are among the most advanced general-purpose conversational agents available. Furthermore, new models such as DeepSeek-R1 (debuted in January 2025) have emerged, claiming comparable performance to ChatGPT at a reduced cost (10). Domain-specific variants are also forthcoming; for example, OpenAI's “Dental GPT” (released in June 2024) addresses questions specific to dentistry (11). As these models continually refine their reasoning and training capabilities, their practical functionalities are progressively enhanced. In fact, large language models like ChatGPT-4 and Gemini have demonstrated the ability to comprehend context and deliver sophisticated responses within healthcare settings (12, 13). By providing on-demand medical guidance, AI chatbots can assist individuals in managing their health more effectively, particularly when access to human healthcare providers is limited (6, 14).

Despite their promise, questions persist regarding the accuracy and reliability of AI chatbots within healthcare environments. Several studies have reported commendable performance; for instance, Arpaci et al. (2025) observed that ChatGPT-4 and Gemini provided responses to common oral health inquiries that closely aligned with expert (Fédération Dentaire Internationale - FDI) answers, with ChatGPT-4 demonstrating marginally greater accuracy (15). Similarly, a particular study in the dental field reported that a specialized “Dental GPT” model achieved the highest accuracy for prosthodontics-related questions, whereas ChatGPT-4 scored lower, and DeepSeek generated more comprehensible text (11). Conversely, other research findings have been more critical. Molena et al. (2024) noted that ChatGPT-3.5 occasionally delivered inconsistent or incorrect responses to clinical inquiries, including errors in calculations and citations of non-existent literature (16). These mixed results highlight an ongoing debate: AI chatbots can provide comprehensive health advice, yet instances of errors or “hallucinations” can occur, and the information may not always be current or entirely dependable. Systematic reviews further indicate that evidence regarding the clinical efficacy of patient-facing chatbots remains limited (6, 17).

While comparative evaluations of LLMs in dentistry are emerging, they have largely focused on diagnostic, surgical, or prosthodontic topics (18, 19). A notable gap remains concerning the performance of AI chatbots, including both general-purpose and domain-specific models, in addressing patient inquiries within cosmetic dentistry, a high-demand area where patients frequently seek online information. To the best of our knowledge, no study has directly compared the performance of both leading general-purpose models (ChatGPT-4o, Gemini, DeepSeek) and a newly released domain-specific model (DentalGPT) on a comprehensive set of patient inquiries related to tooth whitening. Recognizing this gap is of considerable importance: tooth whitening remains a prevalent consumer trend, yet the safety and accuracy of online advice—particularly that generated by AI—regarding this procedure are uncertain. Consequently, this study seeks to bridge that gap by directly comparing four leading chatbots—OpenAI's ChatGPT-4o, Google's Gemini, DeepSeek R1, and DentalGPT—in their responses to comprehensive frequently asked patient questions about tooth whitening. This study will generate the most common questions related to tooth whitening using reputable search query tools, followed by a systematic assessment of each chatbot's responses in terms of readability, usefulness, accuracy, and reliability. Through this analysis, this study aims to identify the respective strengths and weaknesses of each AI tool within the context of this popular dental concern, thereby evaluating their capacity to adequately meet patient information needs regarding tooth whitening.

## Materials and methods

### Ethical approval and study protocol

**Figure 1:**
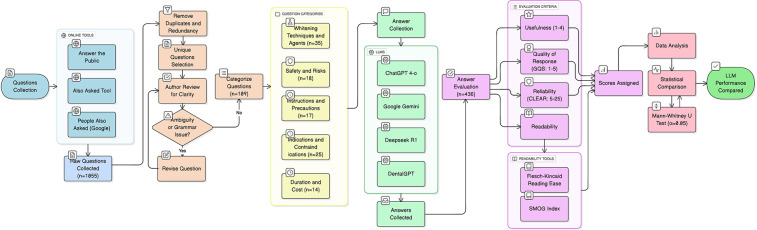
Flowchart illustrating the process of question selection and analysis. An initial pool of 1,055 questions was generated from online tools. After removal of duplicates and irrelevant questions, 109 unique questions were retained, categorized into five domains, and submitted to four LLMs. Their responses were then evaluated for usefulness, quality, reliability, and readability.

Ethical approval was not required for this study, as it did not involve human participants, animal subjects, or any access to private patient data. The research was conducted exclusively through the analysis of publicly accessible artificial intelligence models, using a set of simulated patient questions. The study protocol, detailing the transparent process of question generation, selection, and analysis, is provided in Figure 1.

### Generation of simulated patient questions for LLMs

A total of 1,055 patient-like questions were generated using online query tools (“Also Asked,” “Answer the Public,” and Google's “People Also Ask”) with the keywords “tooth whitening” and “dental bleaching”. All questions were open-ended and formulated to simulate realistic patient scenarios. The initial list was compiled into a single Excel worksheet (Microsoft, Redmond, WA) and independently reviewed by two authors (A.H., Y.H.). Duplicates, ambiguous queries, and questions lacking sufficient detail were removed, resulting in a final list of 109 questions. These questions were reviewed for grammatical accuracy and clarity prior to submission to the large language models (LLMs).

The questions were subsequently categorized into five subgroups (Whitening Techniques and Agents (*n* = 35), Safety and Risks (*n* = 18), Instructions and Precautions (*n* = 17), Indications and Contraindications (*n* = 25), Duration and Cost (*n* = 14)) to evaluate the key aspects relevant to patients interested in or undergoing tooth whitening, Table 1.

**Table 1:** Selected sample of patient questions across evaluation domains.

Category of Tooth Whitening Questions	Question Number and Body
Techniques and Agents	Q1) What is dental bleaching?
	Q9) Dose any over the counter teeth whiteners work?
	Q22) Is hydrogen peroxide safe for your teeth?
	Q28) Can I mix hydrogen peroxide with toothpaste?
	Q35) Is teeth whitening foam effective?
Safety and Risks	Q39) What is the safest concentration of hydroden peroxide to use while doing home bleaching?
	Q41) Why teeth whitening cause sensitivity?
	Q44) Does bleaching your teeth hurt your gums?
	Q49) What are the risks of internal teeth whitening?
Instructions and Precautions	Q55) Can I sleep with teeth bleach on?
	Q63) Should I use fluoride after whitening?
	Q67) Can I drink coffee or tea after teeth whitening?
	Q68) How to keep teeth white after professional whitening?
Indications and Contraindications	Q81) Can children do teeth whitening?
	Q85) Can I whiten dental restorations?
	Q93) Will teeth whitening remove white spots?
Duration and Cost	Q98) How long does teeth bleaching last?
	Q103) How long do teeth pores stay open after whitening?

This table presents representative examples of the simulated patient inquiries used to assess the large language models, highlighting common concerns within each category related to tooth whitening procedures.

### Collection and processing of LLMs responses

The 109 finalized questions were submitted to ChatGPT-4o, Gemini, DeepSeek R1, and Dental GPT within two consecutive days, using the same laptop connected to a wired internet connection to ensure consistency. Separate accounts were created for each platform, and the “New Chat” function was used for every question to minimize potential retention bias. The “regenerate response” option was disabled to ensure a single consistent answer from each model. Responses were independently collected by one author [O A] and stored in 4 separate, anonymized Word documents (A, B, C, and D) to maintain evaluation blindness.

A representative sample question, along with the corresponding full-length responses from all four LLMs, is provided in Supplementary Table S1 to illustrate the nature and variation of the data collected.

### Assessment of LLMs' responses

The answers were independently assessed by 2 board-certified specialists trained in using standardized assessment tools to minimize bias. Prior to the main assessment, both evaluators underwent a calibration session using a pilot set of 20 questions and answers not included in the study to ensure a consistent understanding and application of the assessment tools. Any discrepancies in evaluations were resolved through discussion with a third expert to reach a consensus before assessing all AI chatbot responses. This consensus-based approach ensured a unified and reliable final score for each response. The assessment process was focused on the following four key aspects, outlined in Table 2:

1. Usefulness: To measure the usefulness of ChatGPT responses with regard to tooth whitening and dental bleaching, a modification of a previously used scale to grade ChatGPT responses was adopted in the present study (20). The scale was devised to grade the information generated by ChatGPT into 4 scores: (1) Very useful (provides a comprehensive and correct information that are similar to an answer that a specialist in the subject would give); (2) Useful (provides accurate information but missing some vital information); (3) Partially useful (provides some correct and some incorrect information); and (4) Not useful (provides incorrect or misleading information). evaluated on a four-point scale
2. Quality of answers: The quality of ChatGPT responses was assessed using the Global Quality Scale (GQS) (21).
3. Reliability of answers: The reliability of responses was evaluated using the CLEAR (Completeness; Lack of false information; Evidence support; Appropriateness; and Relevance) tool (22). This is a recently validated tool that was conceived to standardize the assessment of AI-based model output by giving each point a score on a scale from 1–5 (i.e., 1 = poor; 2 = fair; 3 = good; 4 = very good; 5 = excellent). The overall CLEAR score ranges from 5–25 and is divided into three categories: scores 5–11 are considered “poor content”; scores 12–18 are considered “average content”; and scores 19–25 are considered “very good” content.
4. Readability was assessed using the Flesch-Kincaid Reading Ease (FRE) Score and the Simple Measure of Gobbledygook (SMOG) index, both calculated using an online tool (https://readable.com/readability).

**Table 2:** Assessment tools and scoring criteria for evaluating AI-generated responses on tooth whitening.

Assessment Aspect	Tool/Scale Name	Scoring Range and Interperation
Usefulness	Modified 4-point scale	Range: 1–4
		1: Very useful (provides comprehensive and correct information).
		2: Useful (provides accurate information, but missing some vital information).
		3: Partially useful (provides some correct information but also provides some incorrect and misleading information).
		4: Not useful.
Quality of Health Information	The Global Quality Score (GQS)	Range: 1–5
		1: Poor quality, flow, and missing information and not useful.
		5: Excellent quality, flow, and usefulness
Reliability	CLEAR Tool: (Completeness, Lack of false information, Evidence-supported, Appropriateness and Relevance)	Categry Range: 1–5 each
		(1 = Poor, 2 = Fair, 3 = Good, 4 = Very good, 5 = Excellent)
		Ovarall Range: 5–25
		5–11: Poor content
		12–18: Average content
		19–25: Very good content
Readability:	FRE score (Flesch-Kincaid Reading Ease score)	Range: 0–100
		0–10: Extremely difficult to read
		90–100: Very easy to read.
	SMOG index (Simple Measure Of Gobbledygook index)	Approximates the U.S. grade level needed to understand the text.
		6–7: Fairy easy.
		8–9: Moderately easy
		10–12: Moderately difficult
		13+: Challenging to read.

This table summarizes the standardized instruments and modified scales used to quantitatively assess the usefulness, quality, reliability, and readability of the responses provided by the four large language models.

### Statistical analysis

Data was compiled in Excel (Microsoft, Redmond, WA) and analyzed using IBM SPSS Statistics for Windows, Version 29.0 (IBM Corp., Armonk, NY). The mean values of assessment scores for each language model were compared across question categories. Given the nonnormal distribution of the data, nonparametric statistical tests were applied. The Mann–Whitney *U* test was used for comparisons. Given the exploratory nature of the analysis and the absence of primary significant findings, adjustments for multiple comparisons were not applied. Exact *p*-values are reported where applicable. *P*-values below 0.05 were considered statistically significant.

## Results

### Search output and question categorization

A total of 109 questions were subjected to further analysis (Figure 1). The questions were categorized into the following five subgroups: techniques and agents (32.1%, 35 questions), safety and risks (16.5%, 18 questions), instructions and precautions (15.6%, 17 questions), indications and contraindications (23%, 25 questions), duration and cost (12.8%, 14 questions).

### Usefulness of AI chatbots responses

The majority of responses across all language models were rated as “very useful” (68%, *n* = 75). The mean usefulness score was 1.24 ± 0.3 on a scale where 1 represents “very useful” and 4 indicates “not useful”. No responses were classified as “not useful”. Usefulness scores remained consistently high across all language models, with no statistically significant differences observed between them (*p* > 0.05, Table 3), nor across the five question domains (*p* > 0.05, Table 3).

**Table 3:** Usefulness of LLMs at answering patients' related questions about tooth whitening. .

Usefulness score (1–4)	ChatGPT	Gemini	DeepSeek	DentalGPT	*p*-value
*Techniques and Agents*	1.1 ± 0.5	1.31 ± 0.31	1.09 ± 0.19	1.22 ± 0.78	>0.05
Safety and Risks	1.4 ± 0.2	1.24 ± 0.2	1.41 ± 0.25	1.35 ± 0.54	>0.05
Instructions and Precautions	1.03 ± 0.13	1.08 ± 0.12	1.23 ± 0.31	1.2 ± 0.37	>0.05
Indications and Contraindications	1.53 ± 0.4	1.32 ± 0.1	1.51 ± 0.42	1.44 ± 0.62	>0.05
Duration and Costs	1.02 ± 0.3	1.1 ± 0.5	1.07 ± 0.52	1.09 ± 0.72	>0.05
Overall Usefulness	1.216 ± 0.5	1.21 ± 0.6	1.262 ± 0.37	1.26 ± 0.19	>0.05

### Readability

The overall mean Flesch-Kincaid Reading Ease (FRE) score was 36.32% ± 21.9 (range: 8–79), which corresponds to a “difficult” reading level. The mean SMOG index was 10.97 ± 7.4 (range: 5.8–17.3), indicating a reading comprehension level equivalent to approximately the 11th grade. No statistically significant differences in readability scores were observed between different language models (*p* > 0.05, Table 4) or across topic domains (*p* > 0.05, Table 4).

**Table 4:** Readability (FRE scores and SMOG indices) of LLMs at answering patients' related questions about tooth whitening.

FRE score/SMOG index		ChatGPT	Gemini	DeepSeek	DentalGPT	*p*-value
Techniques and Agents	FRE	42.1 ± 23.7	39.34 ± 19.1	36.98 ± 17.2	36.98 ± 17.2	0.57
	SMOG	13.1 ± 8.3	12.4 ± 6.5	13.5 ± 7.4	11.5 ± 9.7	
Safety and Risks	FRE	35.4 ± 19.8	37.2 ± 18.3	34.7 ± 13.5	34.7 ± 13.5	0.22
	SMOG	9.8 ± 5.7	11.3 ± 8.6	10.27 ± 6.4	10.27 ± 8.1	
Instructions and Precautions	FRE	36.9 ± 26.2	32.9 ± 21.4	33.9 ± 20.4	33.9 ± 20.4	0.33
	SMOG	11.2 ± 9.12	10.1 ± 8.1	12.01 ± 6.8	12.01 ± 4.9	
Indications and Contraindications	FRE	33.9 ± 17.2	37.5 ± 23.4	33.1 ± 16.3	33.1 ± 16.3	0.23
	SMOG	10.8 ± 5.9	9.1 ± 7.1	11.1 ± 5.1	8.9 ± 4.9	
Duration and Costs	FRE	40.7 ± 19.4	43.2 ± 21.1	38.9 ± 22.7	38.9 ± 22.7	0.39
	SMOG	12.3 ± 7.3	10.3 ± 8.2	9.79 ± 5.6	9.79 ± 7.3	
Overall readability	FRE	37.8 ± 22.7	38.02 ± 17.8	35.5 ± 18.3	33.96 ± 16.1	0.43
	SMOG	11.44 ± 6.1	10.64 ± 4.8	11.33 ± 4.7	10.49 ± 6.3	

### Quality and reliability

The overall mean Global Quality Scale (GQS) was 3.88 ± 1.96 (range from 1–5), with 42.3% (*n* = 46) of responses achieving the maximum score of 5. Reliability assessment using the CLEAR tool showed a high mean score of 22.5 ± 2.4 (range: 10–25), with 92.6% (*n* = 101) of responses classified as having “very good” content. No significant differences were observed in GQS or CLEAR scores between different question domains or language models (*p* > 0.05, Table 5).

**Table 5:** Quality (GQS) and reliability (CLEAR score) of LLMs at answering patients' related questions about tooth whitening.

GQS/CLEAR Score		ChatGPT	Gemini	DeepSeek	DentalGPT	*p*-value
Techniques and Agents	GQS	3.1 ± 2.37	4.7 ± 2.7	3.01 ± 1.86	3.63 ± 1.44	0.19
	CLEAR	23.1 ± 8.3	19.4 ± 4.3	19.5 ± 7.09	18.15 ± 9.17	
Safety and Risks	GQS	4.7 ± 1.8	3.72 ± 2.6	4.07 ± 1.54	3.47 ± 2.8	0.23
	CLEAR	19.8 ± 8.7	17.24 ± 6.8	24.17 ± 7.4	20.56 ± 9.1	
Instructions and Precautions	GQS	4.9 ± 2.6	3.23 ± 1.98	3.9 ± 2.06	3.39 ± 2.6	0.42
	CLEAR	18.2 ± 9.5	21.31 ± 9.01	22.09 ± 6.1	22.01 ± 3.9	
Indications and Contraindications	GQS	3.1 ± 1.71	4.01 ± 2.43	3.32 ± 2.54	4.18 ± 1.65	0.19
	CLEAR	20.8 ± 7.8	19.01 ± 7.8	21.11 ± 6.1	18.9 ± 7.9	
Duration and Costs	GQS	3.71 ± 1.98	3.9 ± 2.18	4.71 ± 2.35	4.9 ± 2.09	0.53
	CLEAR	23.3 ± 7.01	23.2 ± 8.3	19.19 ± 5.6	19.88 ± 7.3	
Overall Quality and Readability	GQS	3.9 ± 2.7	3.92 ± 1.8	3.8 ± 1.67	3.91 ± 2.1	0.17
	CLEAR	21.04 ± 8.01	20.04 ± 7.8	21.21 ± 5.7	19.9 ± 6.9	

### Performance across question domains

Consistent with the overall findings, a sub-analysis comparing performance scores (usefulness, quality, reliability) across the five question domains also revealed no statistically significant differences (*p* > 0.05 for all inter-domain comparisons). This indicates that the LLMs performed consistently well regardless of whether the topic was clinical (e.g., Safety and Risks) or logistical (e.g., Duration and Cost).

## Discussion

This study provides a systematic comparison of multiple large language models—ChatGPT-4o, Gemini, DeepSeek R1, and DentalGPT—in generating responses to patient-centered inquiries about tooth whitening. Overall, our findings indicate that these AI chatbots are capable of producing highly useful, reliable, and largely high-quality information for patients seeking guidance on this popular cosmetic dental procedure. Importantly, the vast majority of responses were rated as “very useful,” with no instances of misleading or “not useful” answers. These results underscore the potential of LLMs to support patient education and complement professional dental counseling in cosmetic dentistry.

The consistently high usefulness scores observed in this study align with prior evaluations of LLMs in oral health, where ChatGPT-4 and Gemini demonstrated strong accuracy in general dental and medical domains (23–29). Unlike earlier studies reporting occasional inaccuracies, hallucinations, or incomplete responses (6, 30–33), the responses generated across all four models in this study were generally accurate, coherent, and relevant. One possible explanation is the more standardized and well-documented nature of tooth whitening procedures compared to complex diagnostic or treatment scenarios in dentistry, which may reduce the likelihood of factual errors.

A primary finding, however, is that the readability of this high-quality information poses a significant barrier. Our results demonstrate that readability levels, were moderate to challenging for the average reader. A Flesch Reading Ease (FRE) score of 36 is classified as “difficult to read,” and a SMOG index of 11 corresponds to a reading grade level of a high school junior or senior. This is substantially higher than the recommended 6th to 8th-grade level for public health materials, which may severely limit accessibility for populations with lower health literacy and potentially exacerbate health disparities. This issue is not isolated to dentistry (18, 34–36); the finding that AI-generated health information is often pitched at an inappropriately high reading level is consistent with observations in other medical fields, including oncology, cardiology, and ophthalmology (30, 32, 37–39). This recurring theme across specialties highlights a systemic challenge for LLMs in healthcare communication. Future research should, therefore, test specific interventional prompts, such as “Explain this in plain language suitable for a 12-year-old,” to determine if LLMs can consistently generate accurate information at an appropriate readability level.

In terms of quality and reliability, the models performed strongly, with nearly half of all responses receiving the highest Global Quality Scale (GQS) rating and the majority classified as “very good” under the CLEAR tool. The high performance across all models suggests that advances in training strategies and reinforcement learning from human feedback have significantly enhanced the clinical reliability of chatbot outputs (40, 41).

Interestingly, despite its domain-specific training, DentalGPT did not significantly outperform the general-purpose models. This may be because tooth whitening is a well-documented, standardized procedure with a vast amount of high-quality information available in the general corpus used to train models like ChatGPT-4o and Gemini. Consequently, general-purpose LLMs can access and synthesize this information effectively. The advantages of a domain-specific model might be more pronounced in highly specialized, niche, or rapidly evolving areas of dentistry where the general corpus is sparse or less reliable. This finding suggests that for common cosmetic procedures, the development of specialized models may offer diminishing returns compared to continual improvement of general-purpose foundations. Furthermore, the consistent performance across all five domains—from technical clinical questions to those about cost and duration—is encouraging. It suggests that these LLMs are robust tools for providing a wide range of information relevant to a patient's decision-making journey, not just the biological or procedural aspects.

Although the results are promising, certain limitations should be acknowledged. First, the study evaluated responses generated in September 2025, and chatbot performance may evolve rapidly with future updates and retraining. Second, while the models produced accurate and reliable answers, they do not replace the nuanced, individualized guidance of a dental professional. For example, patient-specific factors such as pre-existing sensitivity, enamel defects, or expectations regarding whitening outcomes require clinical evaluation that chatbots cannot yet replicate. Third, the study focused exclusively on English-language queries and responses, which limits the generalizability of our findings to non-English speaking populations. Finally, we did not test the reproducibility of the responses, which can vary between sessions or with slight prompt modifications. The consistency of LLM outputs over time is an important area for future research.

Despite these limitations, the findings highlight the growing role of AI chatbots in supplementing patient education in cosmetic dentistry. Tooth whitening is a high-demand procedure where patients often seek information online prior to consulting a dentist. By providing readily accessible, evidence-informed, and generally reliable guidance, LLMs may help dispel misconceptions, promote safe practices, and enhance patient understanding of the risks and benefits of whitening treatments. However, to optimize their utility, future developments should focus on improving readability and tailoring responses to different health literacy levels.

## Conclusions

In conclusion, ChatGPT-4o, Gemini, DeepSeek R1, and DentalGPT each demonstrated strong potential as patient education tools for tooth whitening. Their outputs were consistently useful, reliable, and of high quality, though often delivered at a reading level above that of the general population. While these AI tools should not replace professional dental advice, they represent valuable adjuncts for enhancing patient access to accurate and timely information in cosmetic dentistry. Future work should focus on improving readability and directly evaluating how AI-generated information influences patient understanding, decision-making, and clinical outcomes.

## Data Availability

The raw data supporting the conclusions of this article will be made available by the authors, without undue reservation.
