# Supplementary material for: The evaluation of tooth whitening from a perspective of artificial intelligence: a comparative analytical study
Source: Front Digit Health. 2025 Nov 24;7:1710159. doi: 10.3389/fdgth.2025.1710159 (PMC12683524; doi:10.3389/fdgth.2025.1710159)
Supplement: Supplementary file 1 [file Table1.docx]

Supplementary TABLE S1: Representative example of LLM responses to a single patient question.

| Question | ChatGPT 4o | Gemini | DeepSeek R1 | DentalGPT |
| --- | --- | --- | --- | --- |
| How long after whitening can I brush my teeth? | After teeth whitening, it's generally recommended to wait at least **30 minutes to an hour** before brushing your teeth. This is because the enamel may be temporarily softened due to the whitening process, and brushing too soon could cause enamel wear or irritation to the gums.  It's also a good idea to use a soft toothbrush and a toothpaste that's gentle on the enamel post-whitening. If you're using any over-the-counter whitening products, always follow the manufacturer's instructions for the best results. | The wait time after whitening depends on the type of whitening treatment you had: Whitening strips: It's recommended to wait at least 30 minutes before brushing your teeth after using whitening strips Professional whitening: After a professional teeth whitening appointment, dentists recommend waiting at least 4 hours before brushing your teeth. In both cases, waiting allows the whitening agents to fully work and minimize tooth sensitivity. Brushing too soon after whitening could irritate your gums or make your teeth more sensitive.Here are some additional tips: Regardless of the whitening method, always follow the manufacturer's instructions or your dentist's advice. When you do brush after whitening, use a soft-bristled brush and brush gently. | After teeth whitening (whether professional or at-home treatments), it's generally recommended to wait at least 30 minutes to 1 hour before brushing your teeth. Here's why:   1. Enamel Softening – Whitening agents (like hydrogen or carbamide peroxide) can temporarily soften tooth enamel. Brushing too soon may wear it down. 2. Sensitivity Risk – Your teeth may be more sensitive post-whitening, and brushing immediately could irritate them. 3. Optimal Results – Waiting allows the whitening agents to fully settle and the enamel to reharden.  Additional Tips:  - Use a soft-bristled toothbrush and a non-abrasive toothpaste (avoid whitening toothpaste for 24–48 hours). - Rinse your mouth with water after whitening to remove residual gel. - Avoid acidic foods/drinks (like citrus or soda) for a few hours.   If you used a professional in-office whitening treatment, your dentist may advise waiting longer (e.g., 2–4 hours). Always follow their specific instructions | After whitening your teeth, it's generally recommended to wait at least **30 minutes to an hour** before brushing. This allows the enamel to re-harden and for any potential sensitivity to subside. Immediately after whitening, your teeth may be more porous and sensitive, and brushing right away can potentially cause irritation or discomfort.To avoid sensitivity, use a soft-bristled toothbrush and a toothpaste designed for sensitive teeth. If you feel discomfort or irritation after whitening, you might want to wait longer before brushing or use a desensitizing toothpaste. |

This table presents the full, unedited responses from ChatGPT-4o, Gemini, DeepSeek R1, and DentalGPT to the question: "How long after whitening can I brush my teeth?". This example illustrates the variations in response length, structure, detail, and complexity that were quantitatively assessed in the main analysis.
